# Comparison of ^68^Ga-PSMA-11 PET/CT with ^11^C-acetate PET/CT in re-staging of prostate cancer relapse

**DOI:** 10.1038/s41598-020-61910-6

**Published:** 2020-03-19

**Authors:** Naresh Regula, Vasileios Kostaras, Silvia Johansson, Carlos Trampal, Elin Lindström, Mark Lubberink, Irina Velikyan, Jens Sörensen

**Affiliations:** 1 Division of Radiology and Nuclear Medicine, Department of Surgical Sciences, Uppsala University, Uppsala, Sweden; 2 Division of Oncology, Department of Immunology, Genetics and Pathology, Uppsala University Hospital, Uppsala, Sweden; 3 Department of Medical Physics, Uppsala University Hospital, Uppsala, Sweden; 4 Department of Medical imaging, Uppsala University Hospital, Uppsala, Sweden

**Keywords:** Cancer imaging, Urological cancer, Prostate, Oncology

## Abstract

Positron emission tomography (PET) imaging is used to localize recurrent disease in prostate cancer (PCa). The tracer ^68^Ga-PSMA-11 visualizes lesions overexpressing prostate-specific membrane antigen (PSMA), while ^11^C-acetate visualizes lesions with increased anabolic metabolism. The aim of this study was to compare the performance of PSMA-PET and acetate-PET in re-staging patients with biochemical relapse. Thirty PCa patients with prostate-specific antigen (PSA) relapse after primary curative therapy were prospectively evaluated. PET/CT examinations using ^11^C-acetate and ^68^Ga-PSMA-11 were performed. Identified lesions were categorized according to anatomical location and PET measurements were correlated with PSA at time of scan. Tumour lesions showed higher semi-quantitative uptake values on PSMA-PET than acetate-PET. PSMA-PET identified more lesions in 11 patients, fewer lesions in eight patients, and identical number of lesions in 11 patients. This study indicates better diagnostic performance of PSMA-PET, particularly in detecting lymph node (81% vs 60%, p = 0.02) and bone metastasis (95% vs 61%, p = 0.0001) compared to acetate-PET. However, 38% of PSMA-expressing metastases appear to be metabolically inactive and 15% of metabolically active metastases lack PSMA expression. Addition of PET with a metabolic tracer, such as ^11^C-acetate, might be beneficial before making treatment decisions.

## Introduction

Prostate cancer (PCa) is the second most frequent cancer and the fifth leading cause of cancer deaths in men worldwide^1^. Early detection of PCa recurrence is of utmost clinical relevance in terms of prognosis^2^. The decision of follow-up treatment in biochemical PCa relapse cases depends on location and spread of the disease. Prostate-specific antigen (PSA) is a protein secreted by the prostate gland and a rise in PSA is detected by analysing venous blood sample collected from suspected PCa patients. PSA is established as a serum marker of PCa and is useful to monitor therapy response and to detect residual or early recurrence of PCa^3,4^. During the treatment process, PSA decreases and reaches the lowest level after several weeks indicating a good response. Recurrent disease is relatively common and is most often detected by rebounding PSA levels. A commonly used definition of biochemical recurrence is the presence of PSA greater than 2 ng/mL after radiation and 0.2 ng/mL after prostatectomy with a persistent elevation confirmed at follow-up^3,4^.

The role of conventional imaging modalities in the setting of PCa recurrence is limited due to poor detection rates and is a major challenge to date^5,6^. Molecular imaging with positron emission tomography (PET) has emerged as an important imaging technique with pivotal diagnostic value in this cohort of patients. The principle of PET imaging is based on labelling a molecule with a short-lived positron-emitting isotope to non-invasively trace a biological process. The PET scanner captures the three-dimensional location of the molecule when the isotope decays. The PET data is then superimposed on simultaneously acquired x-ray images from computer tomography (CT). Different PET tracers have been developed to trace specific biological processes such as glucose uptake, synthesis of macromolecules or receptor expression. Several metabolic tracers, such as ^11^C-acetate, ^11^C-choline, ^18^F-choline, and ^18^F-FACBC, are used clinically to detect PCa recurrence on PET/CT, but with relatively low sensitivity, especially at low PSA levels^7–12^. Historically, the performance of ^11^C-acetate and ^11^C-, ^18^F-labelled choline were not significantly different in localizing recurrent disease^13^.

At our Institution, ^11^C-acetate PET (acetate-PET) is routinely used for staging and therapy evaluation in PCa patients. Several *in-vitro* and *in-vivo* studies linked PCa aggressiveness to malignant de novo lipogenesis, wherein fatty acid synthase (FASN) is the rate-limiting enzyme^14,15^. Previous studies demonstrated the correlation of ^11^C-acetate uptake with enhanced FASN^15,16^ and Leisser *et al*.^17^ showed that FASN upregulation and expression in PCa was correlated to tumour aggressiveness in terms of ^11^C-acetate accumulation. In a previous work, we showed that ^11^C-acetate accumulation measured with PET/CT was a strong predictor of survival in the setting of PSA relapse after surgery, providing evidence for a quantitative relationship between de novo lipogenesis and early death^18^. In the light of these findings, low acetate accumulation in PCa lesions appears to indicate less aggressive clones.

Recent years have seen increased focus on prostate-specific membrane antigen (PSMA)^19–22^. This type II membrane glycoprotein is significantly overexpressed by nearly all PCa cells when compared to other PSMA expressing tissues such as proximal small intestine, kidney, salivary glands and neovasculature of many solid tumours^23–27^. PSMA overexpression in PCa is often 100- to 1000- fold higher than that of normal tissues. Along with overexpression, rapid internalization and blood clearance make PSMA a highly attractive target for PCa PET imaging^28^. The PSMA protein constitutes intracellular, transmembrane and extracellular domains. The extracellular domain contains a binding site for urea suitable for radioligand labelling. Initial studies with a small-molecule urea-based PSMA tracer, ^68^Ga-PSMA-11, suggests that this novel tracer detects PCa recurrence with higher contrast than currently available tracers of metabolic pathways^29–32^. Target specific accumulation of ^68^Ga-PSMA-11 opens up the possibility for accurate quantification of PSMA expression and consequently the disease progression/regression. From the metabolic point of view, similar information is provided by acetate-PET. So far, no study has been conducted to compare the performance of PSMA-PET with acetate-PET in terms of localizing the recurrent disease status.

This study aimed to evaluate the performance of PSMA-PET and acetate-PET in identifying PCa recurrence in patients after curative therapy.

## Methods

### Patient characteristics

Thirty patients were prospectively included and both PSMA and acetate PET scans were acquired within two weeks in 25 subjects. In five subjects the time interval varied from 22 to 66 days. Therapy was not changed between scans. All patients were referred with suspected progressive disease and having PSA values ranging from 0.36 ng/mL to 240 ng/mL following prior curative treatment (e.g. hormonal therapy, chemotherapy, surgery and/or radiation therapy). All relevant clinical data to evaluate the possible effect of different variables including PSA at time of the scan, PSA doubling time (PSA_DT_), PSA velocity (PSA_Vel_), Gleason score (GS) and age were recorded. Findings from both PET scans were discussed in multidisciplinary conferences and the influence of PET on decisions regarding further treatment was documented.

The study was approved by the regional ethics review board (Dnr. 2017/190). Written informed consent was obtained from all research subjects.

### PET/CT imaging protocol

^68^Ga-PSMA-11 was synthesized locally in accordance to good manufacturing practice (GMP) guidelines using a method previously described by Eder *et al*.^33^ (Supplementary Information). ^11^C-acetate was synthesized according to the method proposed by Le Bars *et al*.^34^ based on original synthesis^35^ with in-house modifications (Supplementary Information).

All PET/CT examinations were performed on a Discovery MI PET/CT system (GE Healthcare, Waukesha, WI) with a spatial resolution of 4 mm at the centre of the field of view and with 2 min acquisition per bed position. First, a CT transmission scan (140 kV, 40–80 mA) without contrast medium was obtained. Emission scans from mid-thigh to skull base were acquired 62 ± 4 min (range 60–78 min) after intravenous administration of 2.0 ± 0.3 MBq/kg (range 1.3–2.9 MBq/kg) of ^68^Ga-PSMA-11 and 10 ± 1 min (range 9–14 min) after intravenous administration of 3.9 ± 0.3 MBq/kg (range 3.0–4.8 MBq/kg) of ^11^C-acetate. A diagnostic thoracoabdominal CT-scan with contrast enhancement was performed directly after acetate-PET.

PSMA-PET images were reconstructed using block-sequential regularized expectation maximization (BSREM) (Q.Clear; GE Healthcare) method with β-value 900^36^. Ordered subsets expectation maximization (OSEM) (VPFX; GE Healthcare) method with 3 iterations, 16 subsets, and a 5-mm Gaussian post-processing filter was used for acetate-PET image reconstruction.

### Image analysis

Hermes Hybridviewer, version 2.0.0 (Hermes Medical Solutions AB, Stockholm, Sweden) was used for PET/CT image analysis. All lesions with pathological uptake and high consensual suspicion for recurrence were identified and defined as local recurrence, pelvic or distant lymph node metastases, or bone metastases. Detection rate (DR) was defined as the ratio of identified lesions on each PET image by the total number of identified lesions found on both PET images.

Standardized uptake values were calculated as: activity in region of interest (Bq/cm3) × body weight (g) $\div\,$ injected dose (Bq). Mean and maximum SUV (SUV_mean,_ SUV_max_) for all lesions were recorded on both scans. Tumour volume was measured over a volume of interest (VOI) generated on PET image by including all spatially connected voxels within a fixed threshold of 40% between SUV_max_ and SUV 3. For positive lesions in proximity to the urinary bladder, the bladder was masked to differentiate boundaries and to draw VOI over PCa lesions. The highest SUV_max_ and tumour volume (TV) of any lesion were recorded. Total tumour volume (TTV) was defined as the sum of all individual tumour volumes in each patient. Total lesion activity (TLA) was defined as, SUV_mean_ × TV and SUV_max_ × TV, for TLA_mean_ and TLA_max_ respectively.

### Statistical analysis

Data were presented as mean ± SD unless otherwise stated. Non-parametric Wilcoxon signed-rank test was used to test for group differences. A two-sided Mann-Whitney test was used to evaluate differences concerning PSA values between groups with and without pathological lesions in both PET scans. McNemar tests were used to compare positive PET findings proportionally both on a patient level and at the level of total number of lesions. Non-parametric Spearman correlation (ρ) tests were used for direct comparison of volumetrics from both PET scans. The associations of PSA-derived indices towards PET volumetrics were tested using univariate regression analysis after log transformation. A two-tailed p-value of <0.05 was considered statistically significant. Statistical analyses were performed on JMP V13 (SAS Institute Inc., Cary, NC) unless otherwise stated.

### Ethics declarations

All procedures performed in studies involving human participants were in accordance with the ethical standards of the institutional and national research committee with the principles of the 1964 Declaration of Helsinki and its later amendments. Ethical approval for this prospective study was obtained from the Regional ethical review board (Dnr. 2017/190). Informed consent was obtained from all individual participants included in the study.

## Results

Patient characteristics were summarized in Table 1. Eight patients (27%) had previously undergone radical prostatectomy and 22 (73%) were treated with prior radiation therapy and/or androgen deprivation therapy. Ten of 30 patients were under active hormonal therapy (duration >10 months) at the time of PET. Remaining patients were not in active treatment. The average age of the patients was 69 ± 8 years (range 51–86, median 70.0) with a mean Gleason score (GS) of 7.3 ± 1.2 (range 5–9, median 7) and a mean PSA level of 28.3 ± 37.7 ng/mL (range 3–160, median 14) at time of diagnosis. Mean PSA measured at the time of the first scan was 23.9 ± 56.6 ng/mL (range 0.36–242, median 5).

**Table 1:** Patient characteristics.

Patient No.	age (years)	initial GS	PSA at diagnosis	PSA at scan	Acetate (MBq)	PSMA (MBq)	time diff. (days)
1*	73	8	5.4	4	368	193	3
2	77	7	5.7	11	321	186	1
3*	76	6	19	207	343	198	1
4	65	6	14	3	474	198	13
5*	70	7	7.8	10.5	352	265	10
6*	69	6	5.7	0.37	257	143	3
7	74	7	8.8	28	409	185	10
8*	63	6	15	242	258	148	3
9	67	7	10	3.1	291	146	66
10	80	9	70	2.6	326	145	65
11	70	8	13	9	408	217	22
12	73	9	71	6.9	342	189	3
13	76	6	49	72	360	155	28
14	56	7	3.3	3.3	347	175	10
15	69	7	21	11	376	189	24
16	62	9	22	7.1	411	188	1
17	76	7	8.9	4.5	340	183	3
18	63	9	24	4.5	365	134	8
19	76	7	160	43	417	151	2
20	86	6	5.6	11	282	129	8
21	60	9	22	5.8	499	193	5
22	75	9	61	4.4	320	109	6
23	57	7	17	0.36	446	173	10
24	59	9	4.9	3	328	186	12
25*	71	7	6.4	1.2	302	168	6
26*	72	5	10	7	387	196	1
27	51	7	26	5	311	167	8
28	65	9	130	1.7	395	193	2
29	71	7	5.6	3.6	322	199	2
30*	68	7	-	0.36	275	165	9

*Indicates patients with prostatectomy as primary treatment. PSA at diagnosis was not retrievable in one patient.

**Figure 1:**
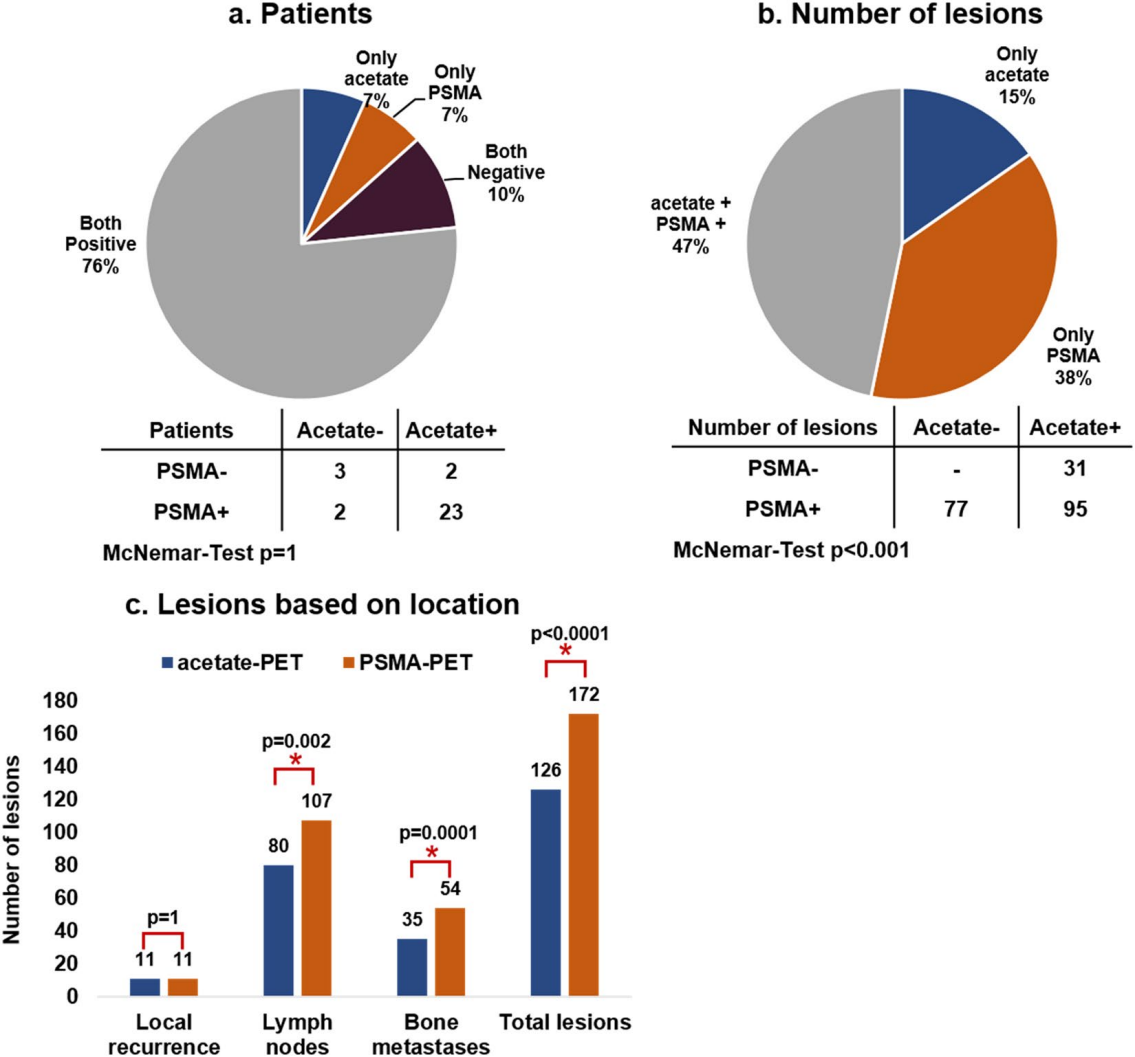
Number and percentage of positive PET on patient level (**a**) and lesion level (**b**) detected by acetate- and PSMA- PET followed by lesions categorized into local recurrence, lymph node and bone metastases (**c**) by both scans.

There were no adverse reactions in any of the patients after injection of either tracer. On the patient level both scans showed at least one lesion highly suspicious of PCa recurrence in 27 of 30 patients (both 83%, p = 1) with an overlap in 23 patients (76%, see Fig. 1a). Three patients with PSA 0.36 ng/mL, 0.36 ng/mL and 0.37 ng/mL at the time of scan showed negative findings on both scans. Mean PSA at time of scan in patients with negative PSMA-PET (n = 5) and acetate-PET (n = 5) was 1.1 ng/mL and 2.3 ng/mL, respectively.

**Figure 2:**
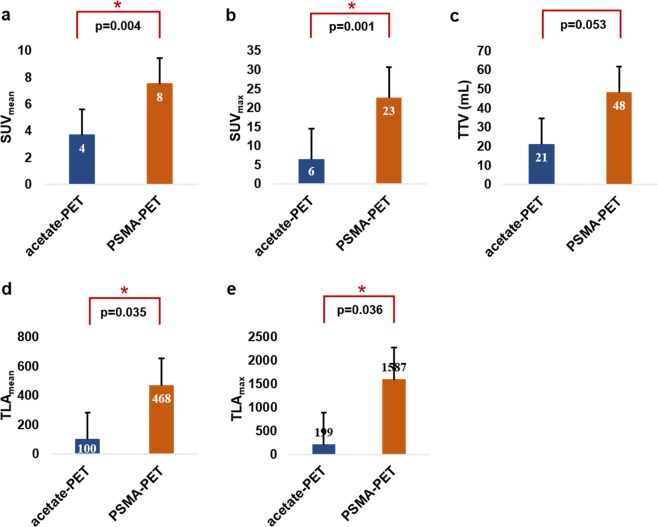
Volumetric parameters derived from VOIs placed over PET images showing average SUV_mean_ (**a**), SUV_max_ (**b**), TTV (**c**), TLA_mean_ (**d**) and TLA_max_ (**e**) on acetate- and PSMA- PET scans.

On the lesion level a total of 203 suspicious lesions were identified, of which 95 lesions (47%) were detected by both scans. PSMA-PET alone identified 77 lesions (38%) while 31 lesions (15%) were only positive on acetate-PET. Intra-lesion comparison showed higher DR for PSMA-PET (85% vs 62%, p < 0.0001) compared to acetate-PET (Fig. 1b,c). In comparison to acetate-PET, PSMA-PET detected identical lesions in 8 subjects, 63 lesions more in 11 patients, and 17 lesions less in 8 patients. Based on the location of PCa lesions (prostatic fossa, pelvic nodes, distal nodes, bone), PSMA-PET showed more wide-spread disease in 9, less in 3, and no change of disease status in 17 patients. PET/CT volumetric parameters are shown in Fig. 2. All volumetrics except TTV were significantly higher on PSMA-PET compared to acetate-PET.

In 13 of 30 patients, local relapse in and around the prostatic fossa was found. Both scans showed local relapse in nine patients, two lesions were detected on PSMA-PET alone, while two showed an increased uptake only in acetate-PET. The lesions of local relapse showed significantly higher uptake for both SUV_mean_ (8.5 ± 6.1 vs 3.8 ± 0.8, p = 0.03) and SUV_max_ (30.4 ± 27.5 vs 6.2 ± 2.9, p = 0.02) on PSMA-PET compared to acetate-PET.

**Figure 3:**
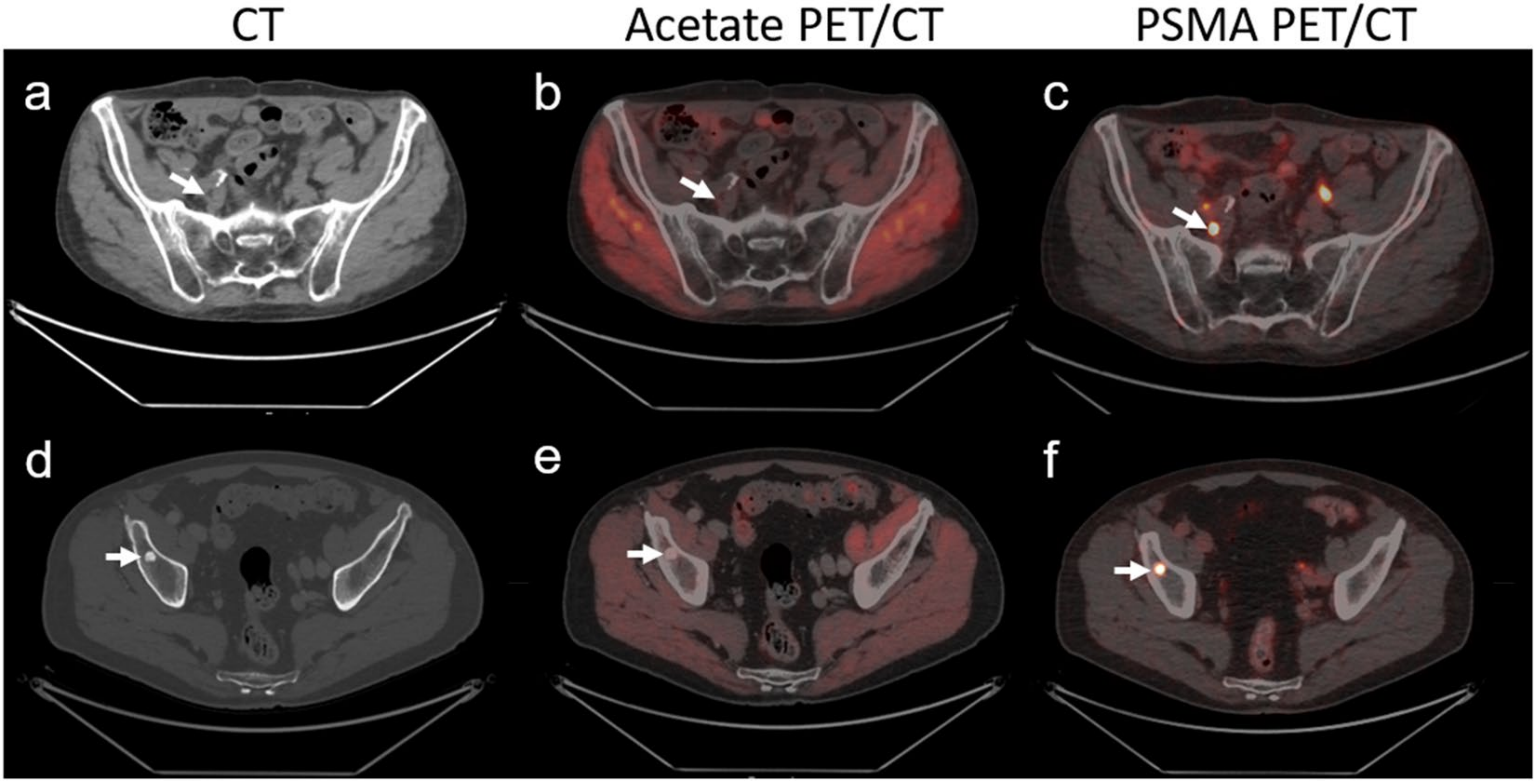
Detection of lymph node (top row) and bone lesions (bottom row) with white arrows on both acetate (**b**,**e**) and PMSA PET (**c**,**f**) along with CT findings (**a**,**d**) in two different subjects. A 76-year-old patient who underwent hormonal and radiation therapy in the past due to PCa (Gleason score 3 + 3). The patient was referred to scans due to PSA elevation 72 ng/mL shows a small lymph node in pelvic region on CT scan a) with no uptake on acetate-PET (**b**) but with positive lesion on PSMA-PET (**c**). A 74-year-old patient referred to scan due to a rise in PSA at 28 ng/mL after curative radiation and hormonal therapy to PCa (Gleason score 3 + 4) showing bone lesion on CT (**d**) with no uptake on acetate-PET (**e**) but positive on PSMA-PET (**f**).

**Figure 4:**
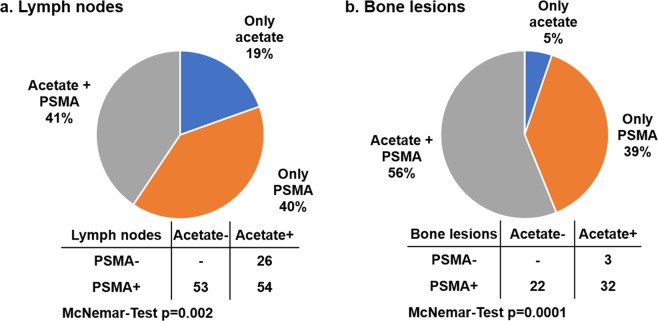
Percentage and number of patients with at least one positive lymph node (**a**) and bone lesion (**b**) on both scans.

Among 16 of 30 patients, a total of 133 lesions suspicious for lymph node metastases were detected (Fig. 3). Of these positive lymph nodes, 53 (40%) were only found in PSMA-PET and 26 (19%) only on acetate-PET. Increased uptake of both tracers was observed in 54 (41%) of positive lymph nodes (Fig. 4). Uptake of ^68^Ga-PSMA-11 was significantly higher than ^11^C-acetate for both SUV_mean_ (6.3 ± 2.5 vs 4.0 ± 0.7, p < 0.001) and SUV_max_ (15.1 ± 10.2 vs 6.3 ± 2.5, p < 0.001) in positive lymph nodes. Consequently, the lymph node DR was significantly higher for PSMA-PET (81%) compared to acetate-PET (60%, p = 0.002).

Bone metastases were detected in 16 of 30 patients (Fig. 3). Out of 57 positive bone lesions, 22 (39%) showed only ^68^Ga-PSMA-11 uptake, while 3 (5%) were only found with acetate-PET (p = 0.0001). Both studies showed pathological uptake in 32 (56%) bone lesions (Fig. 4). As for other lesions, uptake of ^68^Ga-PSMA-11 in bone lesions was also significantly higher for both SUV_mean_ (8.8 ± 6.1 vs 4.4 ± 0.9, p < 0.001) and SUV_max_ (24.1 ± 23.2 vs 7.6 ± 2.8, p < 0.001), compared to ^11^C-acetate uptake.

Specific clinical characteristics of patients with and without pathological findings in PSMA and acetate PET are detailed in Table 2. Between these groups, mean PSA at time of diagnosis was not significantly lower in the group without pathological findings on acetate-PET and PSMA-PET. In contrast, mean PSA at time of scan was significantly lower in the group without pathological uptake both on PSMA-PET (two-sided Mann-Whitney test, p = 0.001) and acetate-PET (p = 0.02).

**Table 2:** Specific characteristics of patients with and without pathological findings on both scans.

Patients	PSA at scan	GS	PSA at diagnosis
with path. findings in acetate-PET (n = 25)	28.2 ± 60.1	7.4 ± 1.2	28.9 ± 38.4
without path. findings in acetate-PET (n = 5)	2.3 ± 2.6	7.2 ± 1	24.8 ± 27.1
with path. findings in PSMA-PET (n = 25)	28.4 ± 60	7.4 ± 1.2	31.3 ± 39.1
without path. findings in PSMA-PET (n = 5)	1.1 ± 1.1	6.8 ± 0.4	9.8 ± 4.5

PSA_DT_ and PSA_Vel_ were available in 28 subjects with mean calculated PSA_DT_ 7.5 ± 7.6 months (range 0.8–27 months, median 4.8 months) and mean PSA_Vel_ 26.1 ± 51.7 ng/mL/year (range 0.1–226.5 ng/mL/year, median of 9.2 ng/mL/year). The DR of acetate and PSMA PET/CT in patients with PSA_DT_ less than 6 months was 81% and 88%, whereas in patients with PSA_DT_ more than 6 months DR of acetate and PSMA PET was 92% and 83%, respectively (p = 0.32). In patients with PSA_Vel_ less than 5 ng/mL/year DR of acetate-PET was 78% compared to PSMA-PET (DR 56%) and in patients with PSA_Vel_ more than 5 ng/mL/year DR was 89% and 100% for acetate and PSMA PET/CT scans respectively.

Univariate analysis showed a significant correlation of PSA_vel_ with all PET parameters from acetate (r > 0.44, p = 0.02) and PSMA (r > 0.51, p = 0.01) PET scans. Similarly, all PET volumetrics except SUV_max_ from both scans showed moderate but significant correlations with PSA at time of scan (Table 3). The correlations of PSA and PSA_vel_ towards PET volumetrics were insignificantly higher for PSMA-PET (p > 0.05 for all comparisons). No significant association of PSA_DT_ towards PET volumetrics was noticed. Comparing the various PET volumetric indices between the two scans directly, there were moderate or good correlations for all (SUV_mean_: ρ = 0.57, SUV_max_: ρ = 0.65, TTV: ρ = 0.77, TLA_mean_: ρ = 0.78, TLA_max_: ρ = 0.79, p < 0.001 for all).

**Table 3:** Univariate analysis of PSA at time of scan and PSA_Vel_ with PET measurements from both acetate and PSMA PET scans after normalizing all variables using log transformation.

PSA at time of scan	r	*p*-value	PSA_Vel_	r	*p*-value
Acetate SUV_mean_	0.41	0.04	Acetate SUV_mean_	0.48	0.02
Acetate SUV_max_	0.37	0.06	Acetate SUV_max_	0.44	0.03
Acetate TTV	0.44	0.03	Acetate TTV	0.46	0.02
Acetate TLA_mean_	0.46	0.02	Acetate TLA_mean_	0.47	0.02
Acetate TLA_max_	0.44	0.03	Acetate TLA_max_	0.46	0.02
PSMA SUV_mean_	0.42	0.04	PSMA SUV_mean_	0.58	0.002
PSMA SUV_max_	0.39	0.05	PSMA SUV_max_	0.53	0.008
PSMA TTV	0.58	0.002	PSMA TTV	0.51	0.01
PSMA TLA_mean_	0.58	0.002	PSMA TLA_mean_	0.57	0.004
PSMA TLA_max_	0.57	0.003	PSMA TLA_max_	0.56	0.004

All the variables showed significant correlation with both PSA at time of scan and PSAVel except the association of SUVmax from acetate and PSMA PET with PSA at time of scan.

Findings from the PSMA-PET scan influenced treatment management in 6 patients (20%) whereas patient management was altered in only one patient based on acetate-PET findings. Oligo-metastatic disease status was defined as the presence of not more than five suspicious lesions on PET scans. Seventeen patients (68%) had oligo-metastatic disease on acetate-PET compared to 14 patients (56%) on PSMA-PET, thus the status was upstaged to metastatic in three patients.

## Discussion

This was a prospective study to evaluate the performance of PSMA-PET compared to acetate-PET in patients with biochemical relapse of PCa after previous curative treatment. The results suggested that PSMA-PET performed better in localizing PCa recurrence in lymph node and bone metastases.

Various metabolic PET tracers reflecting glucose (^18^F-FDG), fatty acid (^11^C-acetate), phospholipid (^11^C-, ^18^F-choline) and amino acid (^18^F-FACBC) pathways are available in PET practice for PCa diagnosis. PSMA-PET was previously shown to detect more lesions than ^18^F-choline^37^, ^11^C-choline^38^ and ^18^F-FACBC^39^. To the best of our knowledge, the current study is the first to compare PSMA-PET to acetate-PET.

Acetate-PET has been used for 15 years at our institution for staging, diagnosing biochemical relapse and treatment guidance in PCa patients. Our previous work emphasized the use of acetate-PET as a prognostic tool to predict survival in patients with PCa recurrence after prostatectomy, in which a highly favourable outcome was seen in subjects with no or minimal pathological ^11^C-acetate uptake even when PSA was well above 1 ng/mL^18^. Since acetate is the major carbon source in PCa, the interpretation of these previous findings was that metastases without visible ^11^C-acetate uptake are minimally aggressive. However, deciding on treatment at relapse ideally requires all tumour deposits to be known for which acetate-PET, like other metabolic tracers, is not a perfect tool. Furthermore, benign lymph nodes are often mildly acetate-avid which can cause false-positive findings. But with our previous experience, most of the benign lesions with mild uptake on acetate-PET and appearance on CT examination, particularly in inguinal and mediastinal regions are identified and discarded. Applying the similar method, diagnostic accuracy of acetate-PET was improved with the specificity optimised to 98%^40,41^.

Recent studies indicate that a positive PSMA-PET can be expected in up to 50% of post-prostatectomy recurrences with PSA lower than 0.5 ng/mL^42^. At this PSA level, pathological uptake is very rare with metabolic tracers, including ^11^C-acetate. We included three patients with PSA below 0.5 ng/mL, and both PET scans were negative in all three. The lowest PSA at which detectable lesions could be seen in this study was 1.7 ng/mL and 1.2 ng/mL for PSMA-PET and acetate-PET, respectively. Because of the small number of patients this sub-analysis is not conclusive.

Clinically, the higher contrast and lesion sensitivity of PSMA-PET resulted in treatment decision changes in six patients when both studies were presented to the multi-disciplinary meetings. In contrast, acetate-PET altered treatment strategy in one subject who had negative findings on PSMA-PET but metabolically active iliac lymph node metastases. Determining the presence of the oligo-metastatic disease is clinically relevant as it influences the treatment strategies. Our study demonstrated that PSMA-PET identified oligo-metastatic disease in fourteen subjects (56%) as compared to 17 patients (68%) with acetate-PET. The findings, however, do not establish the superiority of PSMA-PET for diagnosing oligo-metastatic disease and necessitates a larger study. Nevertheless, similar to other comparative studies with choline PET^31,43^, PSMA-PET detected more bone lesions than acetate-PET. Bone metastatic disease is a strong indicator of poor outcome, further advocating ^68^Ga-PSMA-11 as a most relevant tracer for routine clinical use.

Detection of localized PCa relapse using PSMA-PET (37%) from this study was similar to Eiber *et al*.^42^ (35% of patients). In particular, our lesion-based analysis showed that the majority of patients (69%) with localized relapse within the prostatic fossa were identified by both tracers, while two patients with negative acetate-PET lesions showed ^68^Ga-PSMA-11 uptake and vice versa.

Overall PSMA-PET identified more suspicious lesions compared to acetate-PET in the given cohort. The possible explanations might be due to the presence of metabolically dormant metastases overexpressing PSMA receptors. We used a digital PET scanner with a BSREM reconstruction algorithm allowing full convergence of every single image voxel by controlling image noise providing an increase in signal to noise ratio^36^. ^11^C-acetate data with BSREM was also available for the clinical read but did not result in more findings.

Historically, approximately 10% of PCa clones did not express PSMA receptors^44^. A recent study documented more extreme heterogeneity of PSMA expression in PCa, both on the inter- and intra-individual patient level^45^. High PSMA expression was linked to defective DNA repair, while absent PSMA expression was linked to increased expression of BRCA2 and SOX2, a stem cell pluripotency factor. Heterogeneity was also seen in this study, as 31 lesions in 11 patients were seen with acetate-PET but not with PSMA-PET.

^11^C-acetate uptake reflects the intensity of ongoing de-novo lipogenesis and thus depicts growth, while ^68^Ga-PSMA-11 shows the magnitude of receptor expression. Which type of biological signal that is more relevant for the evaluation of tumour aggressiveness remains unclear. This could be important for choosing therapy in the case of oligo-metastatic disease, but also for therapy evaluation. A retrospective study in castration-resistant PCa patients showed the predictive role of a serial acetate-PET scan during abiraterone treatment in terms of overall and progression-free survival^46^. In our previous study, a negative acetate-PET favoured better outcome in terms of five-year survival in biochemical relapse patients after prostatectomy. In that study, a measurement that combined SUV_max_ and metabolic tumour volume for all detectable lesions (TLA) was the strongest predictor of survival^18^. Similar PET volumetrics can also be applied to PSMA-PET and the current study showed a good correlation of TLA (ρ = 0.79) when the two scans were compared. We also tested the ability of a simple PSA test to predict PET-based tumour burden and found moderate correlations at best (Table 3). We did not find any evidence that the PSA indices could predict the volumetric differences between the PET scans.

Evidence to support PSMA-PET as a tool for treatment response evaluation is limited. So far, only one group presented preliminary results from a retrospective study^47^ and more studies are clearly needed. Moreover, both the issue of lesions with positive PSMA expression but dormant metabolic activity in terms of growth and the fraction of tumours with negative PSMA expression need to be addressed. To study these issues further future studies should consider combining PSMA-PET and a metabolic PET scan.

## Conclusion

In this prospective comparative study, PSMA-PET performed better than acetate-PET for detection of metastatic disease. The study further advocates the use of PSMA-PET as the most relevant molecular imaging method for the initial detection of PCa recurrence. However, PSMA expression is heterogeneous and the addition of PET with a tracer of anabolic metabolism might be needed in patients before therapy decisions.

## Supplementary information

Supplementary information.

## Data Availability

The datasets generated and analysed during the current study are available from the corresponding author on reasonable request.
