# Supplementary material for: Comparison of 68Ga-PSMA-11 PET/CT with 11C-acetate PET/CT in re-staging of prostate cancer relapse
Source: Sci Rep. 2020 Mar 19;10:4993. doi: 10.1038/s41598-020-61910-6 (PMC7081247; doi:10.1038/s41598-020-61910-6)
Supplement: Supplementary file 1 — Supplementary information. [file 41598_2020_61910_MOESM1_ESM.doc]

**Comparison of ^68^Ga-PSMA-11 PET/CT with ^11^C-acetate PET/CT in re-staging of prostate cancer relapse**

Naresh Regula^1^, Vasileios Kostaras^2^, Silvia Johansson^2^, Carlos Trampal^1^, Elin Lindström^1^, Mark Lubberink^1,3^, Irina Velikyan^4^ and Jens Sörensen^1,5^

^1^Division of Radiology and Nuclear Medicine, Department of Surgical Sciences, Uppsala University, Uppsala, Sweden;

^2^Division of Oncology, Department of Immunology, Genetics and Pathology, Uppsala University Hospital, Uppsala, Sweden;

^3^Medical Physics, Uppsala University Hospital, Uppsala, Sweden;

^4^Department of Medicinal Chemistry, Uppsala University, Uppsala, Sweden;

^5^Department of Medical Imaging, Uppsala University Hospital, Uppsala, Sweden

**Corresponding author:**

Naresh Regula, MBBS, PhD student at Uppsala University

E-mail: [naresh.regula@surgsci.uu.se](mailto:naresh.regula@surgsci.uu.se); Tel: [+46765647494](tel://+46765647494)

Submitted to Scientific Reports

## **Automated production of ^68^Ga-PSMA-11**

The active pharmaceutical ingredient starting material PSMA-11 (GMP grade) was purchased from ABX, Germany. The powder was reconstituted in sterile water. Hydrochloric acid (ultrapure, Merck), sodium acetate buffer (pH 4.6, Sigma-Aldrich), sterile water (Fresenius Kabi), sterile saline (Apoteket AB), NaOH (10 M, Sigma-Aldrich), ethanol (APL), water (Fluka, TraceSelect), trifluoroacetic acid (Merck, Darmstadt, Germany) were used as purchased. The starting material, ^68^Ga (t_1/2_ = 68 min, β^+^ = 89%, and EC=11%), was obtained from the pharmaceutical grade ^68^Ge/^68^Ga-generator (1850 MBq, GalliaPharm®, Eckert &Ziegler Radiopharma GmbH, Berlin, Germany) by elution with 0.1 M hydrochloric acid. The appearance of the ^68^Ga eluate was clear and colorless.

The aseptic production of ^68^Ga-PSMA-11 was conducted in a GMP grade A workstation (unidirectional laminar airflow workbench (LAFW)) situated in a cleanroom with GMP grade B air quality. ^68^Ge/^68^Ga generator and Modular-Lab PharmTracer synthesis platform (Eckert & Ziegler Eurotope GmbH, Berlin, Germany) were placed in the LAFW. Sterile, disposable dedicated cassette system (C4-GA68-PSMA) was used. The process included cassette pressure test, labelling synthesis, product purification, formulation, and sterile filtration as well as sterile filter integrity test. The generator eluate was preconcentrated on cation exchange cartridge, and thereafter added to the vial containing precursor in acetate buffer (pH 5). The temperature was optimised (95 °C) to minimize formation of one of the diastereomers. The crude product was purified on a C-8 SPE cartridge. The product was eluted with 50% ethanol, formulated in sterile saline, and passed through a 0.22 µm sterile filter into a sterile 10 mL sterile capped glass bottle. The sterile filter integrity was controlled on the platform applying pressurized air. A sample was taken for the determination of the identity, radiochemical purity, and pH. The total radioactivity of the product was then measured in a dose calibrator.

A high-performance liquid chromatography system (LaChrom, Hitachi, VWR) consisting of an L-2130 pump, UV detector (L-2400), and a radiation flow detector (Bioscan) coupled in series was used for product quality control in terms of chemical and radiochemical purity. Separation of the analytes was accomplished using an analytical column with stationary reversed phase (Chromolith performance RP18e; 100×4.6 mm; particle size: 2 µm). The conditions were as follows: A=10 mM TFA; B=100% acetonitrile (MeCN), 10mM TFA with UV-detection at 220 nm; linear gradient elution: 0 min at 5% B, 0-9 min at 5 to 50% B, 10-11 min at 5% B; flow rate was 2.0 mL/min. Data acquisition and handling were performed using the EZChrom Elite Software Package. The recovery of radioactivity from the analytical column was investigated to confirm that no radioactive product or impurities were left on the column, by collecting HPLC effluent with and without analytical column and measuring the radioactivity. The tests were performed both for the product (^68^Ga-PSMA-11) and free ^68^Ga (III). Specificity, linearity, and precision as repeatability were re-qualified for the UV-detector. The radiochemical purity was defined as the sum of the two diastereomers with the smaller one not exceeding 20% and was higher than 95% with no unknown labelled impurities exceeding 5%.

During the process validation, a sample of the product was kept for subsequent determination of ^68^Ge content, residual solvent content as well as sterility and endotoxin content. The stability of the product at room temperature was monitored by UV-Radio-HPLC for 2 hours.

## **Automated production of ^11^C-acetate**

^11^C-acetate was synthesized according to the method proposed by Le Bars et al. ^1^, ^2^, with modifications and on in-house built automated system (SYNTHIA) routinely used at Uppsala PET center. Briefly, ^11^C was obtained (^11^N (*p*, α) ^11^C) as [^11^C]-CO_2_ on MC-17 MeV cyclotron (Scanditronix). [^11^C]-CO_2_ was trapped and concentrated on a solid support at -190 °C, then released by heating and transferred in a stream of helium to a loop or vial coated with a methyl Grignard reagent. The crude product was purified in-line on a semi-preparative HPLC system using reversed phase column. The quality control was performed on the HPLC system using strong anion exchange analytical column. The radiochemical purity of ACE was higher than 95% and no unknown labelled impurity larger than 5%. The solution was passed through a 0.22 *µ*m filter. The product was supplied in 5-8 mL sterile saline with 1 mL 0.1M phosphate buffer and ethanol (<10% V/V), with pH 7. The total radioactivity of the product was then measured in a dose calibrator.


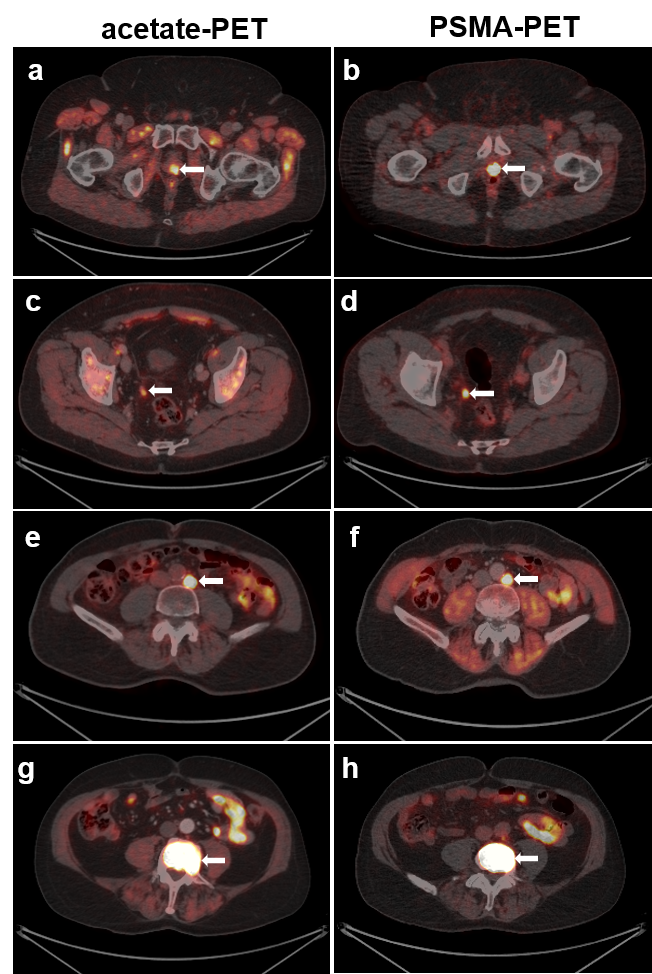


Supplementary Figure 1. Examples of PCa recurrent lesions detected by both acetate-PET (a, c, e and g) and PSMA-PET (b, d, f, and h). a-b) 69-y-old man with Gleason 7 prostate cancer treated with hormonal therapy followed by radiation therapy presented with rising PSA (11 ng/mL) and PSA doubling time of 6.9 months showing local recurrence in prostate fossa on both scans. c-d) 73-y-old man with Gleason 8 prostate cancer treated with radical prostatectomy and salvage radiation who presented with rising PSA level (4 ng/mL) and PSA doubling time of 2.84 months showing positive regional lymph node on both scans. e-f) 86-y-old man with Gleason 6 prostate cancer treated with radiation therapy presented with rising PSA level (11 ng/mL) and PSA doubling time of 6.8 months showing positive para-aortic lymph node on both scans. g-h) 76-y-old man with Gleason 6 prostate cancer treated with radical prostatectomy and salvage radiation followed by hormonal therapy presented with rising PSA level (207 ng/mL) and PSA doubling time of 2.1 months showing positive bone lesion over thoracic vertebra on both scans.

## **Reference**

1. Le Bars, D., Malleval, M., Bonnefoi, F. & Tourvieille, C. Simple synthesis of [1-11C]acetate. *Journal of Labelled Compounds and Radiopharmaceuticals* **49**, 263-267 (2006).

2. Pike, V.W., Eakins, M.N., Allan, R.M. & Selwyn, A.P. Preparation of [1−11C]acetate—An agent for the study of myocardial metabolism by positron emission tomography. *The International Journal of Applied Radiation and Isotopes* **33**, 505-512 (1982).
